# Using the Systemic Immune-Inflammation Index (SII) as a Mid-Treatment Marker for Survival among Patients with Stage-III Locally Advanced Non-Small Cell Lung Cancer (NSCLC)

**DOI:** 10.3390/ijerph17217995

**Published:** 2020-10-30

**Authors:** Tithi Biswas, Kylie H. Kang, Rohin Gawdi, David Bajor, Mitchell Machtay, Charu Jindal, Jimmy T. Efird

**Affiliations:** 1 Department of Radiation Oncology, University Hospitals, Case Western Reserve University, Cleveland, OH 44106, USA; tithi.biswas@uhhospitals.org; 2 Department of Radiation Oncology, Washington University School of Medicine and Alvin J. Siteman Comprehensive Cancer Center, St. Louis, MO 63110, USA; kylie@wustl.edu; 3 Wake Forest School of Medicine, Winston-Salem, NC 27101, USA; rgawdi@wakehealth.edu; 4 Medical Oncology, Seidman Cancer Center, Case Western Reserve University, Cleveland, OH 44106, USA; David.Bajor@uhhospitals.org; 5 Department of Radiation Oncology, Penn State University, Hershey, PA 17033, USA; mxm753@case.edu; 6 Faculty of Science, University of Newcastle, Newcastle 2308, Australia; charujindal@gmail.com; 7 Cooperative Studies Program Epidemiology Center, Health Services Research and Development (DVAHCS/Duke Affiliated Center), Durham, NC 27705, USA

**Keywords:** lung cancer, lymphopenia, neutrophilia, radiation, systemic immune-inflammation index

## Abstract

The Systemic Immune-Inflammation Index (SII) is an important marker of immune function, defined as the product of neutrophil-to-lymphocyte ratio (NLR) and platelet count (P). Higher baseline SII levels have been associated with improved survival in various types of cancers, including lung cancer. Data were obtained from PROCLAIM, a randomized phase III trial comparing two different chemotherapy regimens pemetrexed + cisplatin (PEM) vs. etoposide + cisplatin (ETO), in combination with radiotherapy (RT) for the treatment of stage III non-squamous non-small cell lung cancer (NSCLC). We aimed to determine if SII measured at the mid-treatment window for RT (weeks 3–4) is a significant predictor of survival, and if the effect of PEM vs. ETO differs by quartile (Q) level of SII. Hazard-ratios (HR) for survival were estimated using a proportional hazards model, accounting for the underlying correlated structure of the data. A total of 548 patients were included in our analysis. The median age at baseline was 59 years. Patients were followed for a median of 24 months. Adjusting for age, body mass index, sex, race, and chemotherapy regimen, SII was a significant mid-treatment predictor of both overall (adjusted HR (aHR) = 1.6, *p* < 0.0001; OS) and progression-free (aHR = 1.3, *p* = 0.0072; PFS) survival. Among patients with mid-RT SII values above the median (6.8), those receiving PEM (vs. ETO) had superior OS (*p* = 0.0002) and PFS (*p* = 0.0002). Our secondary analysis suggests that SII is an informative mid-treatment marker of OS and PFS in locally advanced non-squamous NSCLC.

## 1. Introduction

Lung cancer ranks as the leading cause of cancer-related death in the United States and globally [1]. Non-small cell lung cancer (NSCLC) is the most common subtype, accounting for 85–90% of new lung cancer diagnoses in the recent era. About one-third of patients with NSCLC present with locally advanced, non-metastatic disease [2,3]. Even with significant treatment advances, the outcomes for unresectable stage III NSCLC remain poor, with many patients failing to achieve complete response or long-term survival [4]. Only about 14% of stage IIIA and 5% of stage IIIB lung cancer patients survive five years [5]. Thus, there is continued interest in identifying prognostic and predictive factors to improve survival.

In recent years, there has been increased interest in systemic inflammation markers in oncology for their prognostic and predictive potential. Systemic inflammation plays a prominent role in all stages of tumor development and progression, impacting proliferation, survival, metastasis and response to systemic therapies [6]. Continuous exposure to localized inflammatory states within the premalignant tumor microenvironment underlies malignant transformation through intrinsic and extrinsic pathways. Inflammation within these distinct spaces incites genetic mutations, promotes angiogenesis, and increases tumor growth by suppressing anti-tumor immune responses [7].

The localization of inflammatory activity exposes pre-malignant cells to reactive oxygen species (ROS), which in turn induces deleterious mutations to tumor suppressor genes and activates key oncogenes [8]. Malignant cells have also been implicated in the attraction and secretion of pro-inflammatory factors such as IL-1, IL-6, IL-8, and TNFα, which promote further oncogenesis [9]. Specific to NSCLC, mutations on the PTEN tumor suppressor gene triggers upregulation of HIF-1 and HIF-1-dependent transcription of CXCR4, a chemokine receptor gene. This receptor is important in inflammation and cell survival within the tumor microenvironment [10].

Changes in neutrophil, platelet, and lymphocyte counts act as indicators of inflammation and/or impairment of tumor-targeted immune response. Neutrophils are leukocytes with numerous pro-oncogenic properties that operate as critical mediators of localized inflammation in cancer. An increased neutrophil count arises from increased secretion of hematopoietic cytokines, indicative of a more aggressive nature of the tumor and helps to promote tumorigenesis and metastasis [11,12]. IL-1, a leukocyte activation factor increased in inflammatory states, has been shown to raise systemic neutrophil levels (neutrophilia), lower systemic lymphocyte counts (lymphopenia), and traffic neutrophils into regions of localized inflammation [13]. Platelets, the acellular components of megakaryocytes, perform a similar role in promoting tumorigenesis and angiogenesis through pro-inflammatory stimuli. Unlike neutrophils and platelets, increased lymphocyte counts have been associated with tumor suppression, apoptosis of cancer cells, and improved overall survival. Low pre-treatment lymphocyte count alone has been shown to be an unfavorable prognostic factor in NSCLC and is associated with increased lymphatic invasion and recurrence of NSCLC [14,15,16,17,18,19]. Radiotherapy (RT) directly destroys mature circulating lymphocytes at radiation doses as low as <1 Gy, leading to a blunted systemic tumor-targeted immune response [20,21,22,23].

The systemic immune-inflammation index (SII), defined as the product of neutrophil-to-lymphocyte ratio (NLR) and platelet count (P), is a simple and noninvasive pretreatment prognostic indicator of tumor advancement in NSCLC patients [24,25,26,27,28,29,30,31]. The presence of pre-treatment systemic inflammation, vis-à-vis an increased baseline level of SII has been shown to be predictive of clinical outcomes in various cancers including lung cancer [25,32,33,34]. This composite marker uses neutrophil, platelet, and lymphocyte counts to quantify total body inflammation and reflects the balance of host inflammatory and immune status [35]. It is highly reproducible, inexpensive, and widely available as part of routine complete blood count (CBC) measurements, making it a promising prognostic marker. However, it is unclear if SII also holds predictive potential for differentiating the effectiveness of chemotherapy. This question is important for identifying patients who might best benefit from dynamic mid-treatment changes.

In this secondary analysis of data from the PROCLAIM study, we examined the prognostic significance of SII values measured at mid-RT on overall (OS) and progression-free (PFS) survival. We further aimed to test the hypothesis that pemetrexed + cisplatin (PEM) and etoposide + cisplatin (ETO) would have different survival outcomes when stratified by high (above median) and low (below median) values of SII at mid-RT.

## 2. Materials and Methods

### 2.1. Study Design

Data for the current analysis were obtained from the PROCLAIM trial following approval by Eli Lilly [36]. Briefly, PROCLAIM was a randomized open-label phase III clinical trial comparing survival of patients with pathologically confirmed stage IIIA/B unresectable nonsquamous NSCLC, who were administered PEM with concurrent thoracic RT followed by consolidation pemetrexed (arm A) vs. ETO with concurrent thoracic RT followed by non-pemetrexed consolidation (arm B). Patients were eligible if they were ≥18 years old and had an Eastern Cooperative Oncology Group (ECOG) PS of 0/1. They also were required to have adequate organ and pulmonary function and evaluable disease on computed tomography (CT) imaging or a measurable lesion according to Response Evaluation Criteria in Solid Tumors v1.0 [37]. Targeted thoracic radiation doses ranging from 60–66 Gy (2 Gy/fraction daily, 5 days per week) were delivered concurrently with day 1 of chemotherapy. For our analysis, data used included pre-treatment CBC along with differential white count and weekly differential blood count including neutrophil, platelet and lymphocyte count, which were used to compute SII values. Mid-treatment SII was defined as values collected during weeks 3–4 of six weeks of RT, based on the expected nadir value usually seen during this time period.

Among the 598 randomly assigned patients in PROCLAIM (301 arm A; 297 arm B), approximately 7% (18 arm A; 25 arm B) were not treated owing to unmet protocol entry criteria and/or patient/physician decision, leaving 555 patients. Furthermore, those with metastatic disease (remaining in the study after randomization), those with incomplete staging information, demographic details (race, sex), or RT start or key laboratory dates, or those lacking follow-up times were excluded from the current analysis (*n* = 7). Therefore, in our final analysis, 548 patients were included.

### 2.2. Statistical Analyses

Categorical variables were denoted as frequencies and percentages, while continuous variables were reported as medians and interquartile ranges (IQR). SII values were transformed to logarithmic scale to minimize skewness of the underlying distribution and referred to hereafter simply as SII (unless otherwise indicated). Adjusted hazard ratios (aHR) and 95% confidence intervals (CI) were used as the estimated measure of survival risk and were computed using a Cox (proportional hazards) regression model, accounting for the correlated structure of the data (i.e., multiple specific lab values for some patients during the mid-point (week 3–4) time window) [38,39,40]. Demographic variables (age, sex, and race) and treatment were included as model covariates. Models also were adjusted for body mass index as a predisposing factor for inflammation [41].

OS was defined from the date of random assignment (baseline) to any cause of death. PFS was defined from the date of random assignment (baseline) to the first date of documented objective progressive disease or death. OS time was censored at the date the patient was last known to be alive if not dead at the time of data lock. Similarly, PFS time was censored at the date of the last objective progression-free disease assessment. For patients who received subsequent systemic anticancer therapy before disease progression or death, PFS time was censored at the date of the last objective progression-free disease assessment before the date of the subsequent systemic anticancer therapy. To visualize survival times, product-limit estimates and corresponding Kaplan–Meier curves were computed over all observations, regardless of independence [42].

*p*-values for linear trend were computed using a likelihood ratio test [43], while those for interaction were based on the relative effect difference on the logarithmic scale [44]. Unknown values in the predictor variables of a Cox regression model may unduly bias effect estimates if they are not completely missing at random (CMAR). They also may reduce the ability to reject a false null hypothesis even when the values are CMAR. An iterative (multi-stage) expectation-maximization (EM) algorithm with nearest-neighbor adjustment and parametric imputation was used to account for missing baseline and follow-up SII values, respectively [45,46].

The parallel hazards assumption was not violated in our main Cox regression models [47]. SII values were initially analyzed as a continuous variable. Given that values were approximately symmetrical after logarithmic transformation, with homogeneous risk within categories, we then stratified the data by quartiles for the Cox regression analyses [48]. Potential outcome related factors were carefully assessed for over-adjustment bias and unnecessary adjustment by our clinical team prior to inclusion as covariates in our multivariable models [49].

Rounding was performed using the method of Holly and Whittemore [50]. Statistical significance was defined as *p* < 0.05. SAS statistical software (version 9.4, SAS Institute Inc., Cary, NC, USA) was used for all analyses.

## 3. Results

In our analyzed cohort, 548 patients received RT, with the majority being white (72%), male (61%), and presenting with stage IIIB cancer (52%) (Table 1). The median age at baseline was 59 years (IQR = 14). Patients were followed for a median of 24 months (IQR = 23). The baseline and mid-treatment median values were as follows: 1.7 and 0.92 for neutrophil, 0.64 and −0.45 for lymphocyte, 5.7 and 5.4 for platelet, and equally 6.8 and 6.8 for SII, respectively.

**Figure 1:**
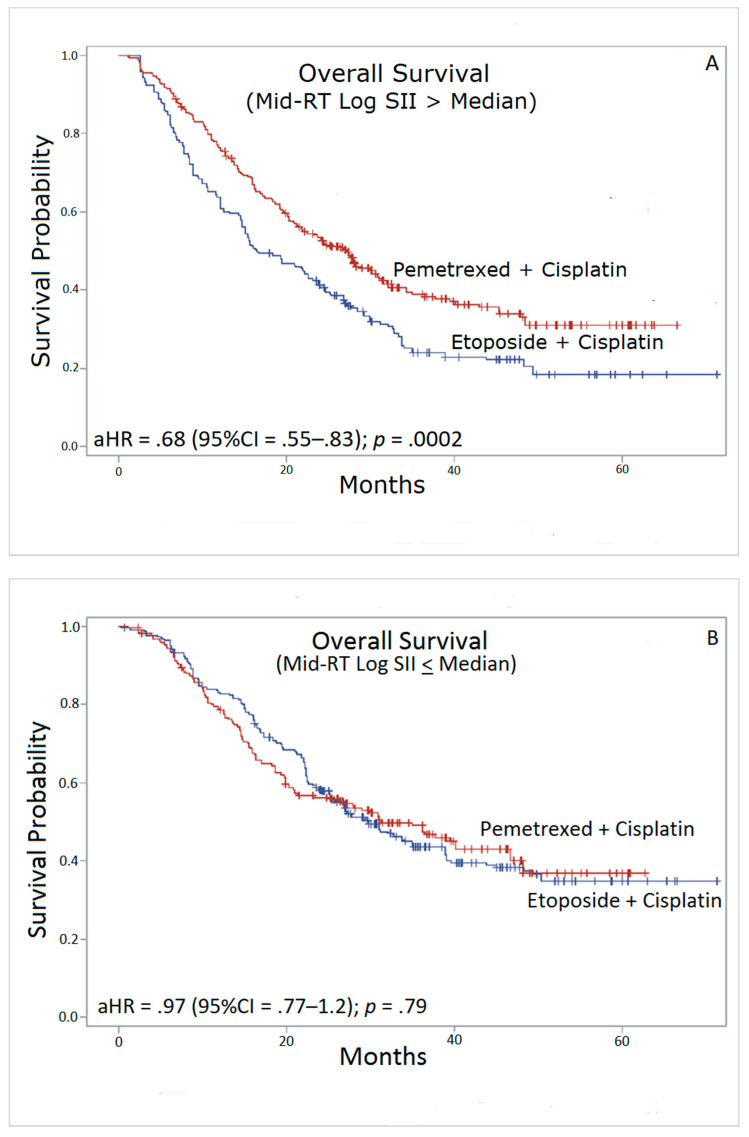
Kaplan–Meier estimates of overall survival (pemetrexed + cisplatin vs. etoposide + cisplatin) for (**A,** top panel) mid-RT log SII > median and (**B**, bottom panel) mid-RT log SII ≤ median. Hazard ratios (HR) and 95% confidence intervals (CI) adjusted for age (years), body mass index (BMI), sex, and race. aHR = Adjusted HR. CI = Confidence interval. SII = Systemic immune-inflammation index.

**Figure 2:**
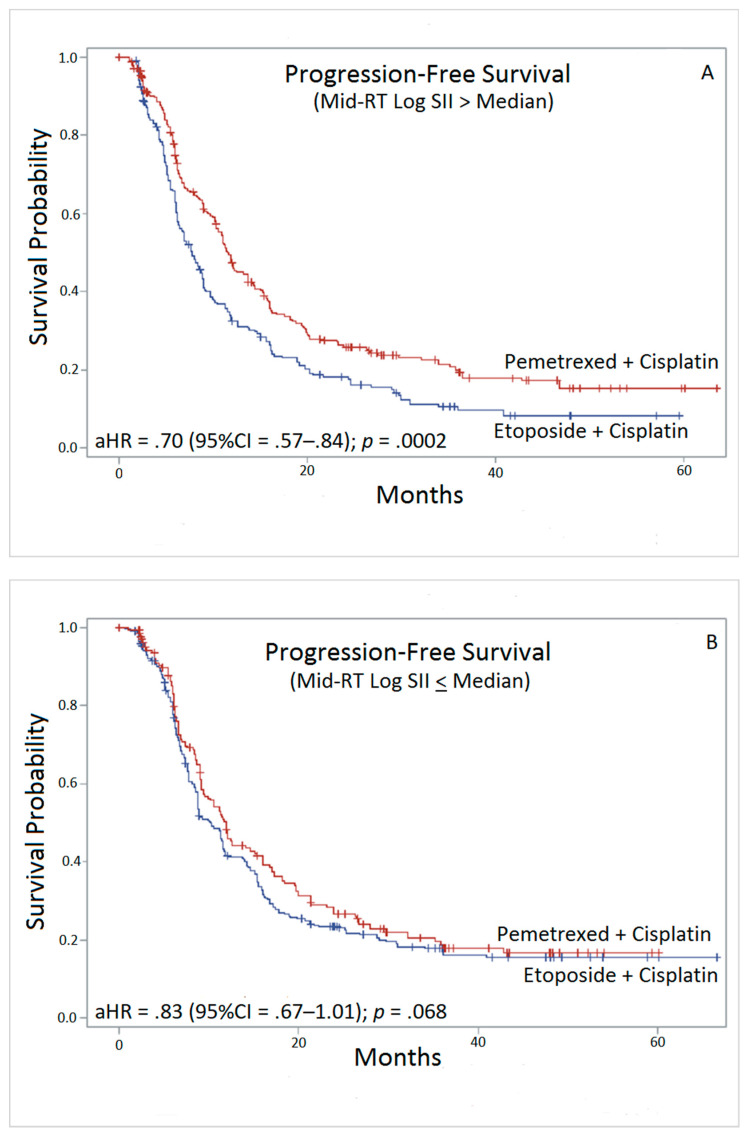
Kaplan-Meier estimates of progression-free survival (pemetrexed + cisplatin vs. etoposide + cisplatin) for (**A**, top panel) mid-RT log SII > median and (**B**, bottom panel) mid-RT log SII ≤ median. Hazard ratios (HR) and 95% confidence intervals (CI) adjusted for age (years), body mass index (BMI), sex, and race. aHR = Adjusted HR. CI = Confidence interval. SII = Systemic immune-inflammation index.

Adjusting for age, BMI, sex, race, and treatment, baseline SII values were statistically significant predictors of survival for the quartile 4 (Q_4_) vs. quartile 1 (Q_1_) (OS: aHR = 1.9, *p* < 0.0001; PFS: aHR = 1.5, *p* = 0.0053). Similarly, at the mid-treatment point, Q_4_ had significantly worse OS and PFS outcomes compared with Q_1_ (OS: aHR = 1.6, *p* < 0.0001; PFS: aHR = 1.3, *p* = 0.0072) (Table 2). Among patients with mid-RT SII values above the median (Q_3,4_), those administered PEM vs. ETO had a 1.5-fold (*p* = 0.0002) and 1.4-fold (*p* = 0.0002) lower aHR of dying or progressing during follow up, respectively (Figure 1A and Figure 2A). Clinically, this corresponds to 21 and 19 fewer patients on average dying or progressing in the PEM arm. In contrast, the comparison of PEM vs. ETO among patients falling in the lower (Q_1,2_) SII stratum was not significantly associated with OS (*p* = 0.79) or PFS (*p* = 0.068), with corresponding *p*-for-interaction values of 0.023 and 0.23 (Figure 1B and Figure 2B).

Stratifying the analysis by patients receiving ETO, those with mid-RT SII values >median vs. ≤median had a 1.7-fold (*p* < 0.0001) and 1.4-fold (*p* = 0.0020) higher aHR of dying or progressing during follow up, respectively. However, aHRs for comparing the above and below median mid-RT values of SII were not statistically significant for either overall (1.2, *p* = 0.13) or progression-free (1.1, *p* = 0.27) survival among those treated with PEM. The corresponding *p*-for-interaction values were 0.020 and 0.20.

While higher mid-RT lymphocyte (log-transformed) values (Q_4_ vs. Q_1_) corresponded to better survival (OS: aHR = 0.78, 95%CI = 0.63–0.97); PFS: aHR = 0.72, 95%CI = 0.59–0.87) (not shown in tables), the directional magnitude of the effect was less than the SII survival disadvantage observed for SII (Q_4_ vs. Q_1_), as indicated by their wider CIs.

## 4. Discussion

Our per-protocol analysis of patients at baseline who subsequently received concurrent chemoradiation is consistent with the intent-to-treat findings of the PROCLAIM study, which did not report an OS or PFS advantage of PEM vs. ETO [36]. However, because the incidence of grade 3 or 4 neutropenia in arm A of the PROCLAIM study was significantly lower than in arm B, we decided to examine survival differences between treatment arms at the mid-point of RT, both adjusting for and stratifying our results by SII (not specified as *a priori* endpoints in the protocol).

We found that patients in this cohort of locally advanced (stage IIIA/B) non-squamous NSCLC experienced tumor progression and died sooner when they had mid-RT SII values falling into the 4th vs. 1st quartile (Q_4_). Specifically, among participants with mid-RT SII values above the median (Q_3,4_), those who received PEM vs. ETO had significantly better OS and PFS. We also observed a significant interaction effect with respect to patients who received ETO. Those in this group progressed and died sooner if they had mid-RT SII values above vs. below the median, in contrast to the PEM arm.

The last two to three decades have seen incremental modifications in RT, surgery, and systemic therapy in the management of stage III NSCLC, with the current definitive treatment typically being a combination of RT and a platinum-based chemotherapy agent, followed by immune checkpoint inhibitor durvalumab. During definitive chemoradiation, the most used chemotherapy regimen in the United States is weekly low-dose paclitaxel and carboplatin. An alternate regimen is another platinum-based doublet like ETO. While a radiation dose of 60 Gy is the standard based on randomized trial results, the preferred concurrent chemotherapy regimen with RT has not yet been established [51]. The multitargeted antifolate agent pemetrexed has been shown to have survival benefit in stage IV adenocarcinoma of the lung [52,53]. In comparison, ETO has a less favorable toxicity profile (e.g., neutropenia, febrile neutropenia, anemia, and alopecia) than PEM doublets [54,55]. Etoposide also is limited in therapeutic use by myelosuppression, particularly neutropenia [56]. However, a therapeutic benefit for PEM (compared to ETO) was not observed for stage III non-squamous NSCLC based on the recent PROCLAIM study [36,57,58]. Although not completely understood, especially given the complex and interacting effect of RT on neutrophils, lymphocytes, and platelets, it is plausible that a differential pharmacodynamic and survival profile exists between these two doublets, especially with respect to the systemic inflammatory state.

Presently, there are no established prognostic markers aside from standard clinical information for differentiating treatment in stage III NSCLC. During definitive treatment, patients undergo serial CBCs to confirm adequate blood cell counts for continuation of treatment. While this blood work contains a wealth of data regularly collected as a part of the treatment protocol, their prognostic potential has not yet been fully realized. Inflammation in the body triggers lymphopenia, neutrophilia, and thrombocytosis. Platelets bind internal cytokines with proinflammatory growth factors and directly mediate tumorigenesis and angiogenesis [59,60,61,62,63,64,65,66,67]. Lymphocytes, which are reduced by inflammation, typically play a role in destroying and disrupting cancer cell proliferation [68,69,70,71,72]. Neutrophils have been shown to inhibit the T-cell lymphocyte response to tumors and are important in advancing tumor growth and proliferation [73,74,75,76,77]. The mid-RT SII can represent the interactions between these various markers of inflammation in a prognostic and predictive manner, although further studies delving into the inflammatory pathways are needed.

Our observational analysis represents a nominal departure from the intent-to-treat design of the PROCLAIM study and may be prone to residual bias and confounding by factors not adjusted for in our study. However, our covariate adjusted pre-RT baseline results were comparable to those reported by PROCLAIM. While our analysis attempted to account for the correlated structure of the data during the mid-RT window, the possibility exists that the true underlying variance may have been underestimated. An unspecified level of bias also may have been introduced by modeling missing values, although such bias is generally believed to be less than the bias incurred by deleting these data points. In our analysis, we used the median as the cutoff value for SII, given that there is no established uniform cutoff value in literature for this marker [78]. In future studies, a consensus optimal cutoff value for SII should be determined from population-based studies, using appropriate statistical methods to adjust for multiplicity. Furthermore, this was a hypothesis generating analysis and needs further validation with additional studies.

The assessment of other potentially important indicators of tumor progression and survival outcomes in lung cancer patients (e.g., modified Glasgow prognostic score, C-reactive protein to albumin ratio, prognostic nutrition index, pretreatment advanced lung cancer inflammation index, and procalcitonin) were considered to be beyond the scope of the current study but ideally would be included in future comparative analyses [79]. Additionally, our analysis could not account for thymidylate synthase expression, which is known to reduce sensitivity to pemetrexed and time to treatment failure in NCSLC patients [80]. We also did not gauge the extent to which SII may have been differentially influenced by hypercholesterolemia, concurrent infection, or the interaction with various incidental drugs. However, such bias likely is nominal given the randomization of patients in the PROCLAIM trial. An independent data monitoring committee evaluated nonblinded safety information 6 months after the first 100 patients and then after all patients completed consolidation chemotherapy. However, beyond this point, specific information about patient deaths (e.g., autopsy report, exact causes) was not available in our analysis dataset.

The large sample size and systematic collection of data under the auspice of a prospective randomized clinical trial with standardized assessment of laboratory measures across sites are key strengths of this study. An important aspect of randomization is that outcome related factors will tend to be balanced between arms. Our analyses were also less prone to biases that may arise with a single center study.

## 5. Conclusions

The results from this study suggest that the systemic immune-inflammation index (SII) in general is an informative baseline and mid-treatment marker of overall and progression-free survival. Furthermore, we observed a statistically significant interaction of this index with the study drugs at the mid-course of therapy. Further studies are needed to establish its value with the current standard of consolidative immunotherapy following chemoradiation in stage III NSCLC. SII also may serve as a surrogate endpoint in future clinical trials with the advantage of being able to assess the utility of a new investigational compound in terms of months rather than years.

## Figures and Tables

**Table 1:** Baseline (pre-radiotherapy) study characteristics (N = 548) ^‡.^

Characteristic	*n* (%) or Median (IQR)
Age (y)	59 [14]
BMI	25 [5.6]
Sex	
Male	332 (61)
Female	216 (39)
Race	
White	392 (72)
Black	25 (5)
East Asian	115 (21)
Other	16 (3)
Stage	
IIIA	264 (48)
IIIB	284 (52)
Treatment	
Etoposide + Cisplatin (ETO)	268 (49)
Pemetrexed + Cisplatin (PEM)	280 (51)

^‡^ Among patients who received radiotherapy. y = Years. BMI = Body mass index IQR = Interquartile range.

**Table 2:** Multivariable survival models by selected characteristics (N = 548) ^‡^.

Characteristic	Baseline (Pre-RT)		Mid-RT (Weeks 3–4 of RT)	
	OS	PFS	OS	PFS
	aHR (95%CI) ^¥^	aHR (95%CI) ^¥^	aHR (95%CI) ^¥,†^	aHR (95%CI) ^¥,†^
Log (SII): Mean, SD	6.8, 0.76		6.8, 1.0	
Percentile (25, 50, 75)	(6.3, 6.8, 7.2)		(6.1, 6.8, 7.4)	
Q_1_	1.0 Referent	1.0 Referent	1.0 Referent	1.0 Referent
Q_2_	1.0 (0.75–1.5)	1.1 (0.80–1.4)	1.1 (0.86–1.3)	1.1 (0.93–1.4)
Q_3_	1.2 (0.88–1.7)	1.1 (0.84–1.5)	1.4 (1.1–1.8)	1.3 (1.1–1.6)
Q_4_	1.9 (1.4–2.6)	1.5 (1.1–2.1)	1.6 (1.3–2.1)	1.3 (1.1–1.6)
*P*_Trend_^§^	<0.0001	0.0081	<0.0001	0.0024
Age (y)	1.0 (0.9995–1.02)	1.0 (0.995–1.02)	1.01 (1.001–1.02)	1.0 (0.996–1.01)
BMI	0.99 (0.97–1.02)	0.99 (0.97–1.02)	1.0 (0.98–1.02)	0.99 (0.98–1.01)
Sex				
Male	1.0 Referent	1.0 Referent	1.0 Referent	1.0 Referent
Female	0.72 (0.57–0.90)	0.79 (0.64–0.97)	0.71 (0.61–0.83)	0.81 (0.70–0.94)
Race				
White	1.0 Referent	1.0 Referent	1.0 Referent	1.0 Referent
Black	1.2 (0.72–2.1)	1.3 (0.81–2.2)	0.95 (0.63–1.4)	1.2 (0.82–1.7)
East Asian	0.93 (0.69–1.3)	1.3 (1.02–1.7)	0.85 (0.70–1.04)	1.2 (1.05–1.5)
Other	1.1 (0.61–2.2)	1.0 (0.57–1.9)	1.2 (0.73–1.9)	1.0 (0.64–1.6)
Stage				
IIIA	1.0 Referent	1.0 Referent	1.0 Referent	1.0 Referent
IIIB	1.3 (1.02–1.6)	1.3 (1.1–1.6)	1.4 (1.2–1.6)	1.4 (1.2–1.7)
Treatment				
ETO	1.0 Referent	1.0 Referent	1.0 Referent	1.0 Referent
PEM	0.92 (0.74–1.1)	0.83 (0.68–1.01)	0.78 (0.67–0.92)	0.75 (0.65–0.87)

^‡^ Among patients who received RT. ^¥^ Proportional hazard model. ^†^ Accounting for correlated data structure at RT mid-treatment point (weeks 3–4). ^§^ Likelihood ratio test for trend. aHR = Hazard ratio adjusted for variables in column 1. BMI = Body mass index. CI = Confidence interval. ETO = Etoposide + cisplatin. Log = Logarithm. OS = Overall survival. PEM = Pemetrexed + cisplatin. PFS = Progression-free survival. Q = Quartile. RT = Radiotherapy. SII = Systemic immune-inflammation index. SD = Standard deviation. y = Years.
